# Penile Septal Haematoma: A Case Report and Narrative Review of a Rare Urological Condition

**DOI:** 10.7759/cureus.97334

**Published:** 2025-11-20

**Authors:** Stefanos Gkaliamoutsas, Kyungmin Kim, Theodora Stasinou, Benjamin R Grey, Ian Pearce, Anas Hattab, Vaibhav Modgil

**Affiliations:** 1 Department of Urology, Manchester University NHS Foundation Trust, Manchester, GBR; 2 Department of Urology, Manchester Andrology Research Collaborative, Manchester, GBR; 3 Department of Radiology, Manchester University NHS Foundation Trust, Manchester, GBR; 4 Faculty of Biology, Medicine and Health, The University of Manchester, Manchester, GBR

**Keywords:** erectile dysfunction, magnetic resonance imaging (mri), penile haematoma, penile trauma, septal haematoma

## Abstract

Penile septal haematoma is a rare form of penile trauma characterised by localised blood accumulation within the intercorporeal septum, without complete tunica albuginea rupture. Unlike classic penile fractures that present with a ‘popping’ sound, immediate detumescence, and significant swelling, septal haematomas usually manifest more subtly, with penile discomfort, a palpable mass, or delayed curvature. Magnetic resonance imaging (MRI) and ultrasound are crucial in distinguishing septal haematomas from other conditions, including Peyronie’s disease and penile neoplasms. Treatment options range from conservative management to surgical intervention, though there is no established consensus on best practice. This report presents the case of a 34-year-old male with a penile septal haematoma following sexual intercourse, illustrating the diagnostic and management considerations. As high-resolution imaging becomes more widely utilised, earlier detection may lead to improved patient outcomes. However, further research is needed to determine optimal treatment strategies and assess long-term functional outcomes. Recognising penile septal haematoma as a distinct clinical entity can aid in early diagnosis and appropriate management, potentially reducing complications such as fibrosis, penile deformity, and erectile dysfunction. This report also reviews the existing limited literature on penile septal haematoma, highlighting its clinical presentation, diagnostic challenges, and management strategies.

## Introduction

Penile trauma covers a broad range of injuries, ranging from minor contusion to complete amputation [1]. The penis consists of two corpora cavernosa and a single corpus spongiosum. The corpora cavernosa are enclosed by a thick tunica albuginea, a dense fibrous sheath providing rigidity and protection during erection [2]. Injuries can therefore involve vascular, fibrous, or erectile components depending on the mechanism and force applied. Understanding these layers is essential for interpreting clinical and imaging findings in penile trauma. Penile fracture typically results from blunt trauma to an erect penis, often accompanied by an audible ‘popping’ sound, rapid detumescence, and swelling [1]. However, injuries of the intercorporeal septum may present atypically and may lack these hallmark signs, leading to diagnostic uncertainty. In emergency settings, such atypical presentations may be mistaken for soft-tissue contusion, thrombosis, or early Peyronie's disease, underscoring the need for awareness among non-specialists.

Penile septal haematoma is a rare clinical phenomenon characterised by localised blood accumulation within the penile septum [3]. Septal haematomas remain confined to the septal region without complete tunical disruption [4]. While trauma is the most common cause, affected individuals may present with symptoms such as penile pain, curvature, or a palpable mass. Despite its rarity, prompt recognition and appropriate management may be essential to prevent long-term complications, including penile fibrosis and erectile dysfunction [5]. We present a case illustrating the diagnostic features and management of this rare condition.

Portions of this report were previously presented in poster format at the 26th Annual Fall Scientific Meeting of the Sexual Medicine Society of North America (SMSNA) on October 11, 2025.

## Case presentation

A 34-year-old man presented to the emergency department with a penile injury sustained during vaginal intercourse 10 hours earlier. He reported experiencing a horizontal impact to the penis, followed by pain at the mid-shaft. There was no immediate detumescence. However, he ceased intercourse, and the penis became flaccid within five minutes. The pain subsided within half an hour, and he subsequently went to sleep. The following morning, he experienced a morning erection but noted discomfort at the same mid-shaft location. He was able to urinate without difficulty and reported no history of haematuria. He denied penile deviation, ecchymosis, or dysuria.

On examination, there was no bruising of the penis or scrotum. Both inguinal orifices were normal. The proximal third of the penis was of normal calibre, while a subtle widening of the corpora was noted at the mid-shaft, returning to normal calibre distally. The glans appeared normal. 

**Figure 1:**
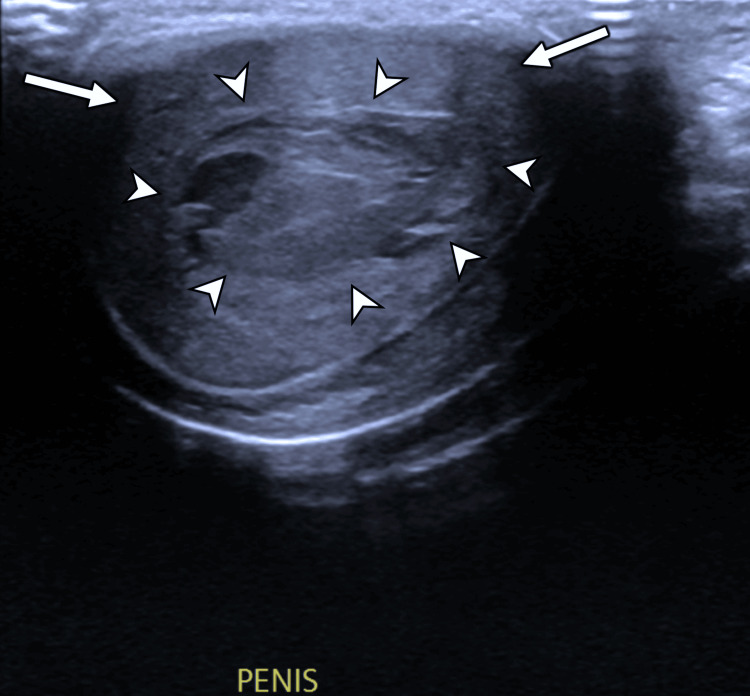
Greyscale ultrasound of the penis in transverse section view Note the central haematoma (white arrowheads) surrounded by penile corpora (white arrows).

**Figure 2:**
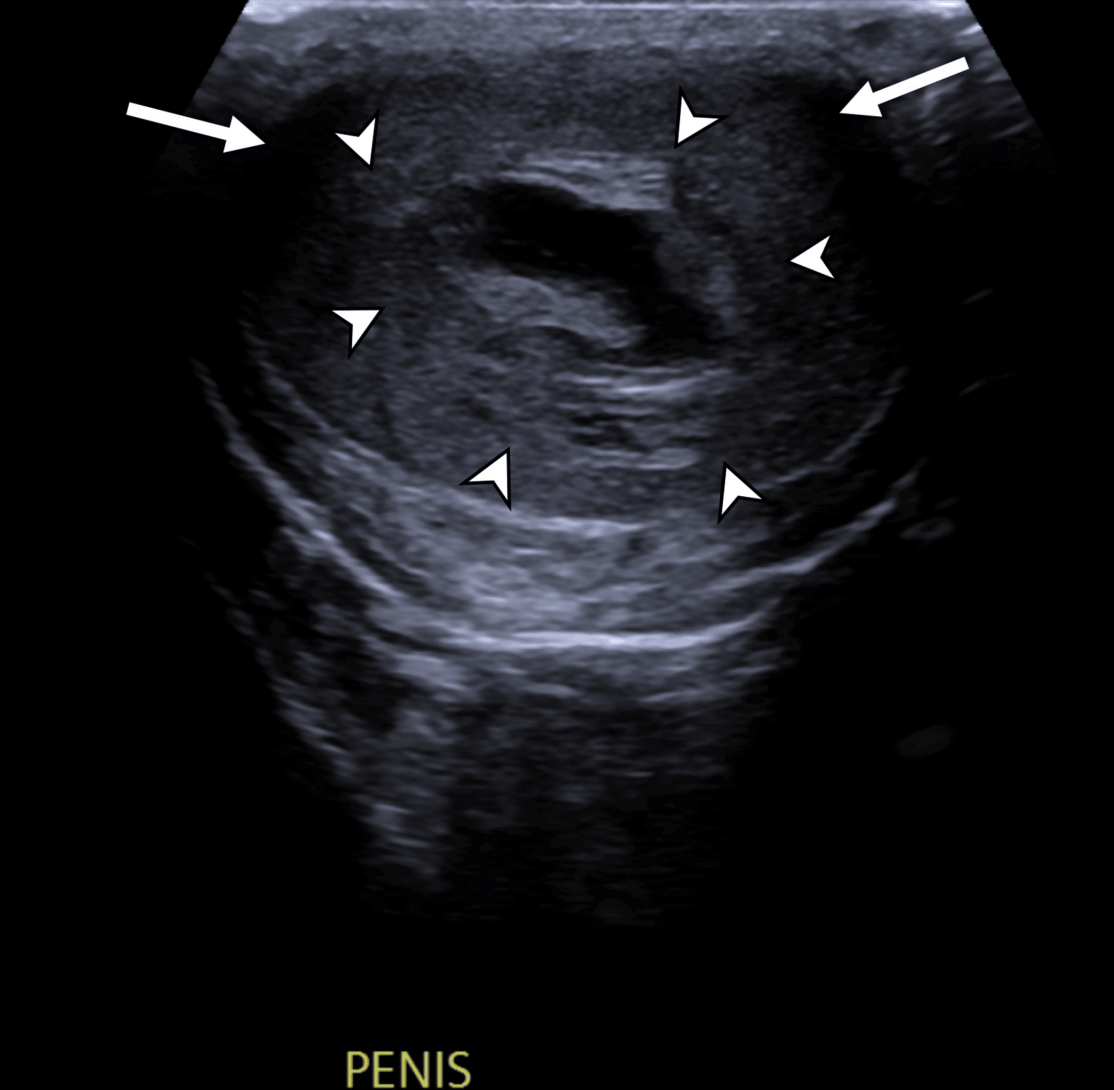
Greyscale ultrasound of the penis in transverse section view Note the central haematoma (white arrowheads) surrounded by penile corpora (white arrows).

**Figure 3:**
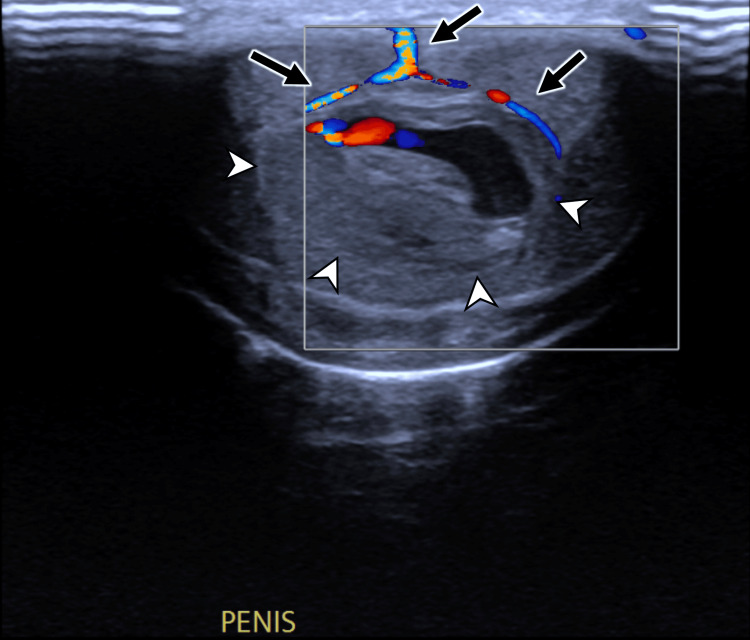
Colour Doppler ultrasound of the penis in transverse section view Note how branches of the dorsal penile vein (black arrows) are splayed by the central haematoma (white arrowheads).

**Figure 4:**
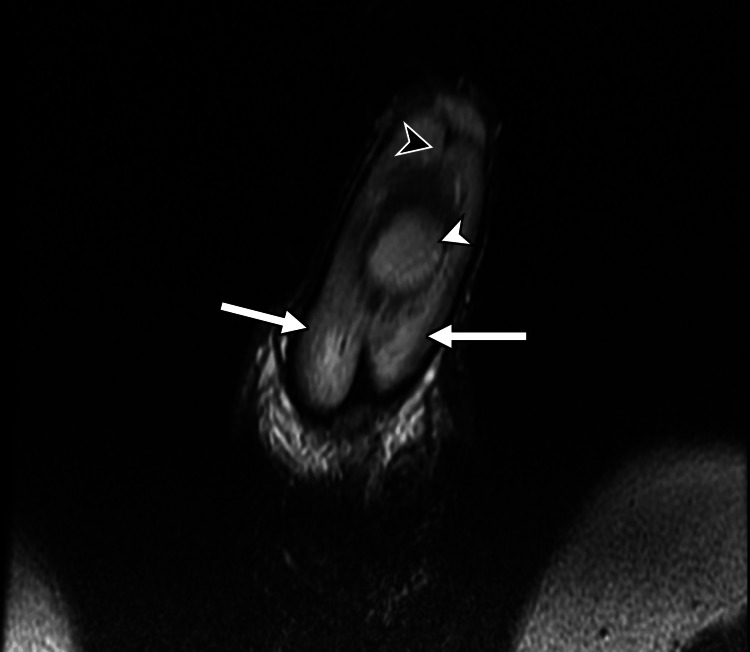
Coronal T2-weighted penile MRI A midline T2-bright collection (white arrowhead) is demonstrated centred over the penile septum (black arrowhead). The penile corpora (white arrows) are surrounding the central septal haematoma.

**Figure 5:**
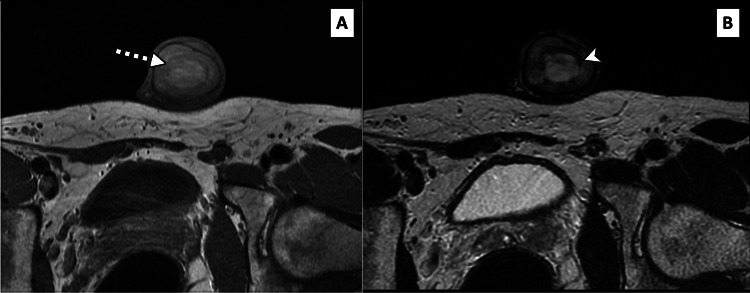
Axial T1-weighted (A) and T2-weighted (B) penile MRI Note how the midline penile collection is both bright on T1 (dashed white arrow) and T2 (white arrowhead), consistent with a penile haematoma.

**Figure 6:**
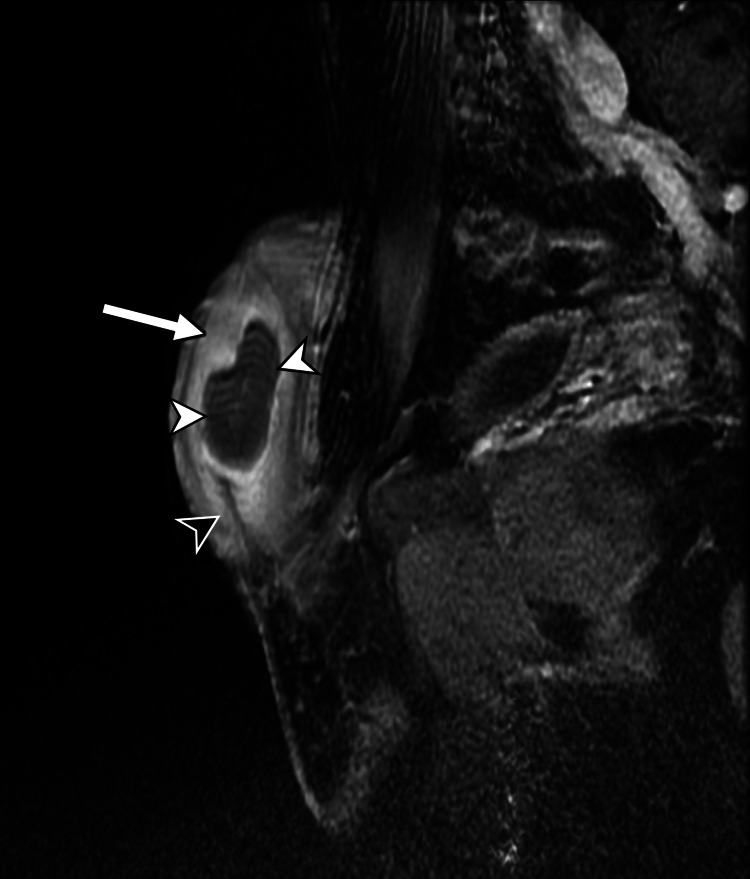
Sagittal fat-suppressed, contrast-enhanced T1-weighted penile MRI The midline haematoma (white arrowhead) is demonstrated centred over the penile septum (black arrowhead). Note how the penile corpora (white arrow) are avidly enhancing, while the central haematoma is not.

An ultrasound scan was performed using a high-frequency transducer with both grey-scale and colour Doppler evaluation. This revealed a 3.5 cm lesion with mixed echogenicity at the mid-shaft, consistent with a septal haematoma (Figures 1-3). Subsequent MRI was performed with axial, coronal, and sagittal T1- and T2-weighted sequences, supplemented by post-contrast fat-suppressed T1 imaging. This confirmed a 4.3 cm midline fluid collection, indicative of septal haematoma and injury, with no evidence of tunica albuginea rupture (Figures 4-6).

Given the preserved erectile function and absence of haematuria or urinary symptoms, conservative management was deemed appropriate with outpatient follow-up arranged. The patient was counselled on the potential risks of fibrosis, erectile dysfunction, and penile curvature. At follow-up at six months, the patient reported no symptoms, no erectile dysfunction, and no penile curvature or deformity. 

The patient's key clinical and imaging characteristics, as well as management and outcome, are summarised in Table 1.

**Table 1:** Clinical and imaging summary of the present case

Parameter	Findings
Age	34 years
Mechanism of injury	Horizontal mid-shaft impact during intercourse
Onset of symptoms	Immediate pain, delayed mild discomfort
Detumescence	Within 5 minutes (no ‘popping’ sound)
Bruising	Absent
Haematuria/Dysuria	Absent
Ultrasound findings	3.5 cm mixed echogenic lesion in septal region; tunica albuginea intact
MRI findings	4.3 cm T1/T2-bright midline collection within septum; no tunical rupture
Management	Conservative
Outcome (at 6 months)	No curvature or erectile dysfunction

## Discussion

Anatomy and pathophysiology of penile septal haematoma

**Figure 7:**
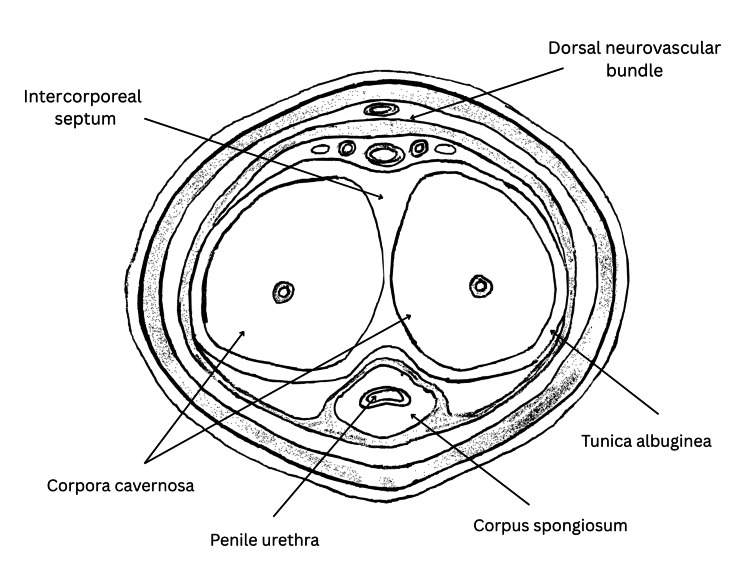
Cross-sectional anatomy of the penis showing the paired corpora cavernosa, corpus spongiosum, tunica albuginea, and intercorporeal septum (typical site of septal haematoma formation). Image Credit: Stefanos Gkaliamoutsas (Author)

The penis consists of three cylindrical structures: the paired corpora cavernosa and the corpus spongiosum, separated by the intercorporeal septum (Figure 7). The septum is formed by the fusion of circular fibres from the tunica albuginea, helping distribute intracavernosal pressure evenly and maintain rigidity during erection [2]. Injury to this fibrous partition can compromise both structural and functional integrity without necessarily breaching the tunica albuginea. Unlike classic penile fractures, septal injuries are confined to the intercorporeal space, producing a contained haematoma rather than full-thickness disruption [4]. This explains why patients may lack the typical 'popping' sound or immediate detumescence of a true fracture.

Localised bleeding within the septum can present as a palpable mass or mild discomfort and, if unresolved, may evolve into fibrosis or calcification, leading to penile curvature or erectile dysfunction [5]. High-resolution imaging modalities, particularly MRI, help delineate these septal lesions and differentiate them from Peyronie’s disease or neoplasms. Understanding the anatomy and biomechanics of the septum is therefore fundamental to distinguishing septal haematomas as a distinct clinical entity, allowing timely diagnosis and management that may prevent long-term complications.

The current case reinforces these observations, showing that, even without tunical rupture, septal injury can result in a well-defined haematoma detectable on both ultrasound and MRI. The complete functional recovery supports prior reports suggesting that early conservative management can yield excellent outcomes when imaging excludes tunical breach.

Literature review

A targeted literature review was conducted using PubMed and Google Scholar databases. The search utilised the following key words: ‘penile trauma’, ‘penile septal haematoma’, ‘penile intracavernous haematoma’, and ‘penile injury’. Inclusion criteria comprised case reports, retrospective studies, and clinical reviews discussing penile septal haematoma. Studies focusing on penile fracture without septal involvement were excluded. 

The review of available literature identified limited but significant cases of penile septal haematoma, highlighting its rarity and the challenges associated with its diagnosis and management. To our knowledge, only two case reports of penile septal haematoma have been described in the literature [4,6], one involving rupture of the penile septum and one without any identified fracture of penile structures. 

A case reported by Turgut et al. detailed a 70-year-old male presenting with a painless penile mass three months after hearing a crepitation sound during coitus [4]. The patient exhibited no erectile dysfunction or penile curvature. Imaging studies, including ultrasound and MRI, revealed a well-defined haematoma within the intercorporeal septum, with no evidence of tunica albuginea rupture. Despite recommendations for surgical drainage, the patient opted for conservative management. Follow-up imaging over a year demonstrated fibrosis and calcification within the septum but no significant functional impairment. 

Another case report by Connolly et al. described a patient diagnosed with penile haematoma after presenting with a six-week history of a growing, non-tender penile lump [6]. Unlike classic penile fractures, this case lacked immediate clinical signs such as rapid swelling or detumescence, contributing to delayed diagnosis. Surgical exploration confirmed septal rupture with haematoma formation, necessitating surgical evaluation and repair. The patient had no functional impairment at follow-up, although post-operative imaging was not performed. 

A study of 650 patients diagnosed with Peyronie’s disease identified 47 cases with septal lesions, including three with cystic lesions [5]. Upon aspiration, these cystic lesions were found to be liquefied haematomas. All three patients had a history of prior sexual trauma and presented with erectile dysfunction. Two patients who sought treatment within two months of their injury experienced a return to normal erectile function after aspiration. However, the third patient, who sustained the injury six months before seeking treatment, did not see any improvement in erectile function. The authors proposed that septal haematomas were secondary to septal fractures and could represent atypical manifestations or precursor lesions to septal fibrosis.

Table 2 summarises previously reported cases of penile septal haematoma, illustrating heterogeneity in presentation, imaging findings, and management strategies.

**Table 2:** Summary of reported cases of penile septal haematoma

Author(s), year	Age (years)	Presentation	Imaging	Management	Outcome
Turgut et al., 2007 [4]	70	Painless penile mass 3 months post-injury	Ultrasound and MRI confirmed septal haematoma	Conservative	Fibrosis, no dysfunction
Connolly et al., 1995 [6]	47	Non-tender lump, 6-week evolution	Ultrasound showed encapsulated deep midline mass; normal retrograde urethrogram	Surgical - septal rupture identified	Full recovery
Brant et al., 2007 [5]	33, 36, and 51	Erectile dysfunction, penile mass. and subjective penile shortening	Ultrasound showed central cystic mass	Aspiration	Early treatment: full recovery; delayed case: persistent erectile dysfunction
Present case	34	Pain after intercourse; no bruising	Ultrasound and MRI: intact tunica, septal haematoma	Conservative	Full recovery

Comparative imaging analysis across cases emphasised the role of MRI in differentiating penile septal haematoma from conditions such as Peyronie’s disease and neoplastic lesions [4,6]. T1- and T2-weighted MRI scans consistently revealed hyperintense haematoma formations, aiding in localisation and staging of the injury. Ultrasound also proved useful for initial assessment, identifying anechoic or hypoechoic septal masses indicative of haematoma [4,6]. Collectively, these cases highlight the diagnostic variability and the absence of standardised management guidelines. 

Clinical presentation

Patients with septal haematomas may present with a variety of symptoms, including a history of penile trauma often associated with a dull ache or deep-seated penile discomfort, a palpable non-tender mass within the penile shaft, no immediate detumescence as seen in classic penile fractures, a gradual increase in penile girth due to progressive haematoma expansion, and difficulty achieving or maintaining an erection [4-6]. Unlike classic penile fractures, where patients experience a sudden loss of erection and severe pain, septal haematomas may have a delayed or subacute presentation. This makes clinical examination alone insufficient for definitive diagnosis, necessitating advanced imaging techniques [4]. 

Diagnostic approaches

The following imaging discussion outlines the roles of ultrasound and MRI in confirming diagnosis and excluding mimics such as Peyronie’s disease or neoplasm. High-frequency ultrasound and MRI are the primary imaging modalities of choice for evaluating penile trauma. Both of these offer detailed anatomical delineation and aid in distinguishing haematomas from other penile lesions [7-9]. 

High-frequency ultrasound is typically the first-line investigation in the assessment of penile trauma, as it is often more easily available than MRI [7,8]. Utilizing a high-frequency linear array transducer (7.5-15 MHz), grey-scale ultrasound commonly demonstrates a well-circumscribed, hypoechoic or heterogeneous lesion confined to the penile septum. Crucially, the tunica albuginea remains intact, unless there is a concurrent penile fracture [5,7,8,10]. On grey-scale imaging, the haematoma appears as a non-vascular soft tissue mass, while colour Doppler imaging shows an absence of internal vascularity, a key feature in differentiating haematomas from penile neoplasms [5,7,8]. In subacute or chronic stages, the septal haematoma may liquefy and may evolve to display an anechoic or complex cystic appearance with internal debris or septations [5,8].

MRI provides superior soft-tissue contrast and is particularly useful in cases requiring further anatomical detail or where sonographic findings are inconclusive [8,9]. Standard MRI protocols should include axial T1- and T2-weighted sequences and at least one coronal or sagittal sequence for multiplanar assessment. Fat-suppressed T1 sequence with gadolinium-based contrast can also be helpful [8,9,11]. Penile septal haematoma presents as a localized area of altered signal intensity within the septum, with its signal characteristics varying according to the age of the haematoma. Acute haematomas typically demonstrate low signal on T2-weighted images and intermediate-to-low signal on T1-weighted images, whereas subacute haematomas often show high signal intensity on both T1- and T2-weighted sequences due to the presence of methaemoglobin [8,9,11]. Post-contrast sequences usually reveal no enhancement within the haematoma itself, which differentiates it from penile neoplasms [8,9]. A distinguishing feature of penile septal haematoma, as compared to penile fracture, is the confinement of the haematoma to the septal region without the disruption of the tunica albuginea or herniation of cavernosal tissue [7-9]. 

Computed tomography (CT) does not offer adequate soft tissue delineation and thus should not be considered in cases of isolated penile trauma. However, in cases of polytrauma (e.g., high-speed road traffic accident) where there is suspicion of concurrent pelvic visceral and/or bony injury, contrast-enhanced CT should be performed [7].

While cavernosography may be used selectively to delineate corporal defects, particularly in cases of suspected penile fracture, it is not performed within the acute setting and does not contribute to the assessment of penile septal haematomas [10,12]. 

Management strategies 

Given the rarity of penile septal haematoma and the lack of literature on the condition, there is no consensus on its management. Options include conservative management, ultrasound-guided aspiration, or surgical intervention, which may be indicated in cases with increasing size, pain, or diagnostic uncertainty. The patient in the case report by Connolly et al. underwent surgical enucleation of an organising haematoma with a fibrotic capsule using a dorsal approach with careful mobilisation of adjacent neurovascular structures, with good functional outcomes [6]. However, in cases of delayed diagnosis, fibrosis may lead to curvature or erectile dysfunction, requiring further intervention [5].

Future directions and research gaps

Despite advancements in imaging, septal haematomas remain underreported. Future studies should focus on long-term functional outcomes following conservative versus surgical management, the role of early MRI screening in suspected cases, and the potential for minimally invasive aspiration techniques to treat larger haematomas without surgery. 

## Conclusions

Penile septal haematoma is a rare but distinct subtype of penile trauma that may present without the classic features of penile fracture. This case, along with limited prior reports, demonstrates that careful clinical evaluation combined with high-resolution ultrasound and MRI allows accurate diagnosis and differentiation from other penile lesions. Conservative management can be effective when tunical integrity is preserved, as illustrated by full recovery at six-month follow-up. Increasing clinician awareness and systematic reporting are essential to define optimal management strategies and long-term outcomes. While broader conclusions remain limited by the small number of published cases, recognition of this entity can help prevent unnecessary surgical intervention and reduce long-term morbidity.
